# A survival analysis approach to determine factors associated with non-retention of newly hired health workers in Iran

**DOI:** 10.1186/s12913-023-09262-5

**Published:** 2023-03-16

**Authors:** Vahid Ghavami, Seyed Saeed Tabatabaee

**Affiliations:** 1 Department of Epidemiology & Biostatistics, School of Health, Mashhad University of Medical Sciences, Mashhad, Iran; 2 Social Determinants of Health Research Center, Mashhad University of Medical Sciences, Mashhad, Iran; 3 Department of Management Sciences and Health Economics, School of Health, Mashhad University of Medical Sciences, Daneshgah avenue, between 16 -18, Faculty of Health, Mashhad, 9137673119 Iran

**Keywords:** Survival analysis, Health workers, Employee, Retention

## Abstract

**Background and aim:**

One of the main tasks of the healthcare human resource management is to maintain and retain professional staff. The high level turnover of professional staff may reduce the quality of healthcare service delivery. Therefore, this study investigated the factors associated with the turnover of the newly recruited healthcare professionals using survival analysis method in Iran.

**Materials and methods:**

This historical cohort analysis comprised 6811 employees who began working at Mashhad University of Medical Sciences between 2005 and 2020. Employees recruited at any of the university’s units between the years 2005 to 2019 were included. We used appropriate descriptive indices and Log-rank test and the Cox proportional-hazards model to assess the staff turnover. A significance level of 0.05 was used for all tests.

**Results:**

The findings of the survival analysis showed that the probability of turnover in one year, two years, and five years of employment were 0.12, 0.16, and 0.27. Based on the findings of the Log-rank test, the probability of turnover in entire of the study period was not statistically different between male and female (p = 0.573), and likewise between employees with healthcare occupations and non-healthcare occupations (p = 0.351). Employees whose current workplace and birthplace were not similar had a significantly higher probability of turnover (p < 0.001). Accordingly, the Cox regression result showed, the risk of turnover for the singles was 1.22 times higher than the married. For the Ph.D degree was 3.23 times higher compared to those with a diploma or an associate degree, and for a bachelor’s or master’s degree was 2.06 times more likely to change their workplace than those with a diploma or an associate degree.

**Conclusion:**

Policies promoting the recruitment of native-born professionals, given priority to the married candidates than single ones, and/or recurring candidates to pledge to stay in the locality of recruitment site can increase the staff retention and reduce the costs of staff turnover including re-hiring, initial and on-the-job training, accommodation, and other extra living consumptions away from home and family.

**Supplementary Information:**

The online version contains supplementary material available at 10.1186/s12913-023-09262-5.

## Introduction

Quality of service delivery in healthcare organizations is significantly influenced by the availability of professional staff [1]. Human resources have long been considered as a critical and strategic factor in the healthcare system. Therefore, ensuring employees’ satisfaction is significantly momentous [2]. Staff job-satisfaction is considered as one of the main drivers for staff retention. Job satisfaction has been defined as ‘the extent to which people like or dislike their job Intrinsic and extrinsic factors of job characteristics influence the level of job satisfaction. Intrinsic factors depend on the characteristics of an employee that include performance, challenges and autonomy, and the extrinsic factors include workload, job security, promotion opportunities and relationships with co-workers [3]. In various studies; association between job satisfaction and staff retention has been reported [4–7]. Staff turnover is one of many indicators used to study job outcomes in the health workforce, retention and intentions are the other indicators which are considered to stay or leave a job. Turnover is defined as an employee leaving a job or organization [8]. Retention is defined that an employee continues the employment and may include a workforce who leaves employment, but remains in the organization in varies positions [9].

Maintaining motivated, skilled and experienced health workers in the right place at the right time is vital to assure the delivery of healthcare services [10]. In the other words, the retention of health workers in healthcare organizations is substantially crucial to the continuation of the provision of healthcare services as well as the development of professional relationships between caregivers and patients, which leads to expected health outcomes[11–13].

In general, the various factors related to turnover of healthcare professionals are categorized into two overall themes: Organizational factors include work environments, organizational culture, commitment, work demands and social support, and individual factors include job satisfaction, burnout and demographic specifications [14]. Various factors are defined as psychosocial factors to the interaction between human resource capacities and the job contents, job structure, management and conditions of organizational environments. Positive interaction between these elements leads to enhanced self-confidence of staff, increased motivation as well as job satisfaction, and on the other hand, negative interaction can deteriorate job satisfaction, poorer life quality and eventually increased to work place staff turnover [15].

Considering the existing shortage of professionals experienced by most health care organizations [16], staff turnover can further exacerbate the gap in quality service delivery [17]. As a matter of fact, one of the key responsibilities of human resource managers is to systematically consider the optimal maintenance of human resources which is paramount importance [18]. Employee turnover is costly for health care organizations [19]. Furthermore, employee turnover reduces the quality and stability of service delivery, and enhance the workload and work-stress among remaining workers [20]. Identifying the underlying factors of employee turnover, avoid adverse repercussions both for service delivery and workplace satisfaction. It makes an incentive based approach for recognizing employee needs to maintain the staff [21].

A cross-sectional study by Tilahun Mekonnen et al. with 427 samples showed that sex, marital status, education, autonomy, and accommodation conditions were found to have a statistically significant association with the intention to leave among health professionals [22]. In Kim et al. study, there was a significant association between age and intent to stay in a job; where older were more likely to remain in the organization. Married employees were substantially more inclined to stay with existing organization than single ones. Likewise, a statistically significant positive association was found between job title and willingness to stay, and employees with related job experience had the substantial intention to stay [23]. Another study showed that intention to stay in an organization was positively correlated with age and job experience [24]. The results of Lulin Zhou et al.‘s study showed that nurses’ job stress factors such as; crucial responsibilities, patients-care workload, interaction with co-workers, daily life issues/conflict, and the lack of professional knowledge and skills have a significant impact on their turnover intention [25]. Shortage of professional employees and high level of turnover are among the biggest challenges in healthcare systems in Iran. Yet, precise statistics on health professional attrition is not available at the national level [26].

Considering the shortage in availability of competent professionals in Iran, and from the other side, staff turnover imposes direct and indirect costs such as; employment process, onboarding and on-the-job training, improper usage of the employment quota license, and being time-consuming process; it is most important for managers in Iran to understand and predict that how long the employees will stay in the organizations and which factors affect this work place turnover. Therefore, this study aims to determine the factors associated with the retention longevity of newly hired employees at Mashhad University of Medical Sciences.

## Method

### Study design and setting

We used a historical cohort study that included 6811 newly hired employees at Mashhad University of Medical Sciences (MUMS) from March 2005 to December 2019 and followed in March 2020. The employees’ status, including replacement, workplace change, occupation, resignation, long-term leave, laying off and leave without payment were investigated. MUMS consists seven schools including; Medical School, Dentistry, Pharmacy, Traditional Persian Medicine, Nursing and Midwifery, Hygiene and Paramedical Sciences, 30 hospitals, and 20 health and treatment networks with a total population of more than 5 million people.

### Study population and sample

The study population included all the employees working in one of the affiliated units of Mashhad University of Medical Sciences. The inclusion criteria was the commencement of staff recruitment at one of the university’s units between 2005 and 2019. Those with employment history to another organization (prior to recruitment at MUMS) were excluded.

### Data collection

The data were collected through a researcher-made checklist that included the demographic profiles of the employees (gender, degree, age, marital status, job title, and experience), the date of employment and the date of turnover. The internal reliability of the checklist was tested by the Cronbach’s alpha coefficient and for all items it was higher than 0.8.

Required information were extracted from the personnel file system keeping the confidentiality rights upon coordination of human resources management, and after the permission of the University Vice Chancellor for Research. Subsequently, based on the statistical of the survival analysis method, factors related to the first turnover/attrition were determined. The start time of the study was the date of employment at MUMS and the end time was the turnover or the last confirmed date of fallow up (March 2020). The endpoint of interest was the time from employment to turnover. Individuals who had not officially requested to turnover by the end of this study (March 2020), death and unavailability were considered as the right censoring.

### Data analysis

Demographic variables, including age, gender, marital status, experience, education, native status, and organizational position were analyzed contextual variables. Based on the statistical analysis, factors related to the first time workplace turnover were retained. In univariate analysis, the Log-rank test was used to compare the personnel probability of retention in comparative gender, married versus single, and native-borne versus non-local groups [27]. Multi-variable analysis, the Cox proportional-hazards model using forward method was employed to simultaneously investigate the association between independent variables and personnel retention [28]. In the Cox regression model, the proportionality assumption was examined and verified using Schoenfeld residuals. Statistical analysis was carried out in R version 4.2.1 using the package “survival”. A significance level of 0.05 was used for all tests.

## Results

In this study, 6811 employees working at Mashhad University of Medical Sciences between the years 2005 to 2020 were investigated. The mean age was 36 ± 6, more than half of them were female (63%), and the majority (93%) was working for healthcare related occupations. The Median follow-up period was 50 months with a minimum and maximum of 1 and 150 months, respectively. 7% of jobs were non-health care and 93% were health care jobs (Table 1). The exact job position of health care center employees can be found in Additional file 1. During the study, the working place of 2105 (31%) employees changed from the employment site, and (69%) were censored (Table 2).

**Table 1:** Demographic features

variable		Numbers	Percentage	Median^*^	P^**^
Gender	Female	4282	63	-	0.573
	Male	2529	37	-	
Marital status	Married	5698	84	-	< 0.001
	single	1113	16	-	
Job category	Non-healthcare	494	7	-	0.351
	Healthcare	6317	93	-	
Education	General practitioner and specialist practitioner and specialized doctorate (Ph.D)	348	5	86	< 0.001
	Diploma and associate degree	1039	15	-	
	Bachelor’s and Master’s	5424	80	-	
workplace and birthplace correspondence	yes	4456	65	-	
	No	2355	35	116	< 0.001

*Median of survival time if it was computable

**Log-Rank test

**Table 2:** Distribution of different reasons for changing the workplace

Type	Number	Percentage
Prolonged absence leading to dismissal	12	1
Resignation	94	4
Permanent transfer	844	40
Temporary transfer	1129	54
Unpaid leave	26	1

Based on the findings of the survival analysis, the probability of turnover for an employee in one year, two years, and five years of employment 0.12, 0.16, and 0.27, respectively. (Table 3).

**Table 3:** Probability of turnover of newly hired health workers in various periods of employment

duration	probability of turnover	95% confidence interval for probability of turnover
Less than 1 year	0.12	0.11–0.13
Less than two years	0.16	0.15–0.17
Less than five years	0.27	0.26–0.29
Less than 10 years	0.40	0.39–0.42

**Fig. 1:**
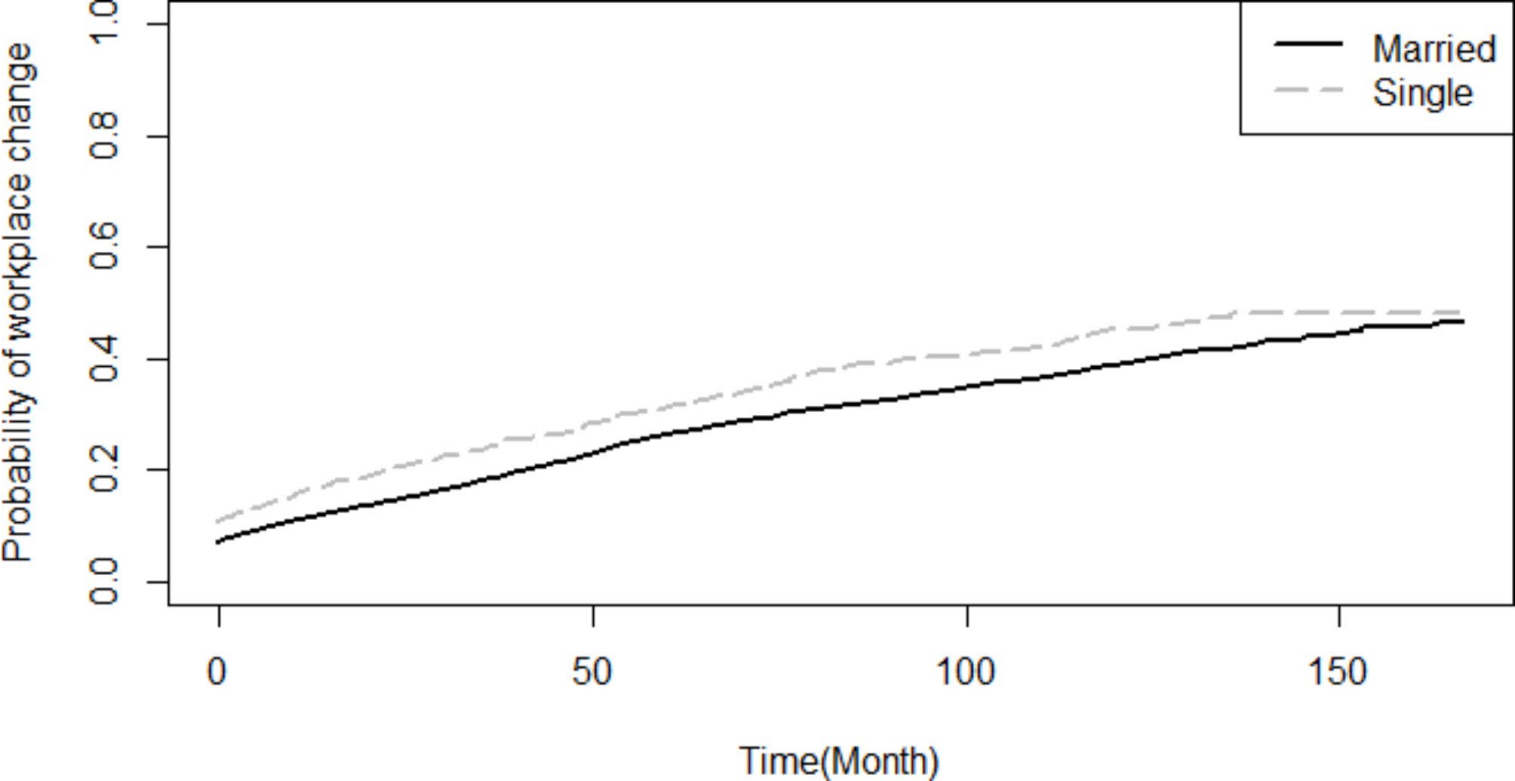
Comparison of the probability of turnover throughout the study period based on marital status

**Fig. 2:**
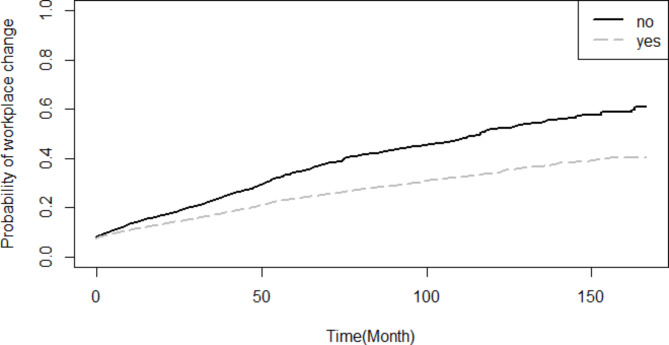
Comparison of the probability of turnover throughout the study period based on equality of birthplace and first workplace

**Fig. 3:**
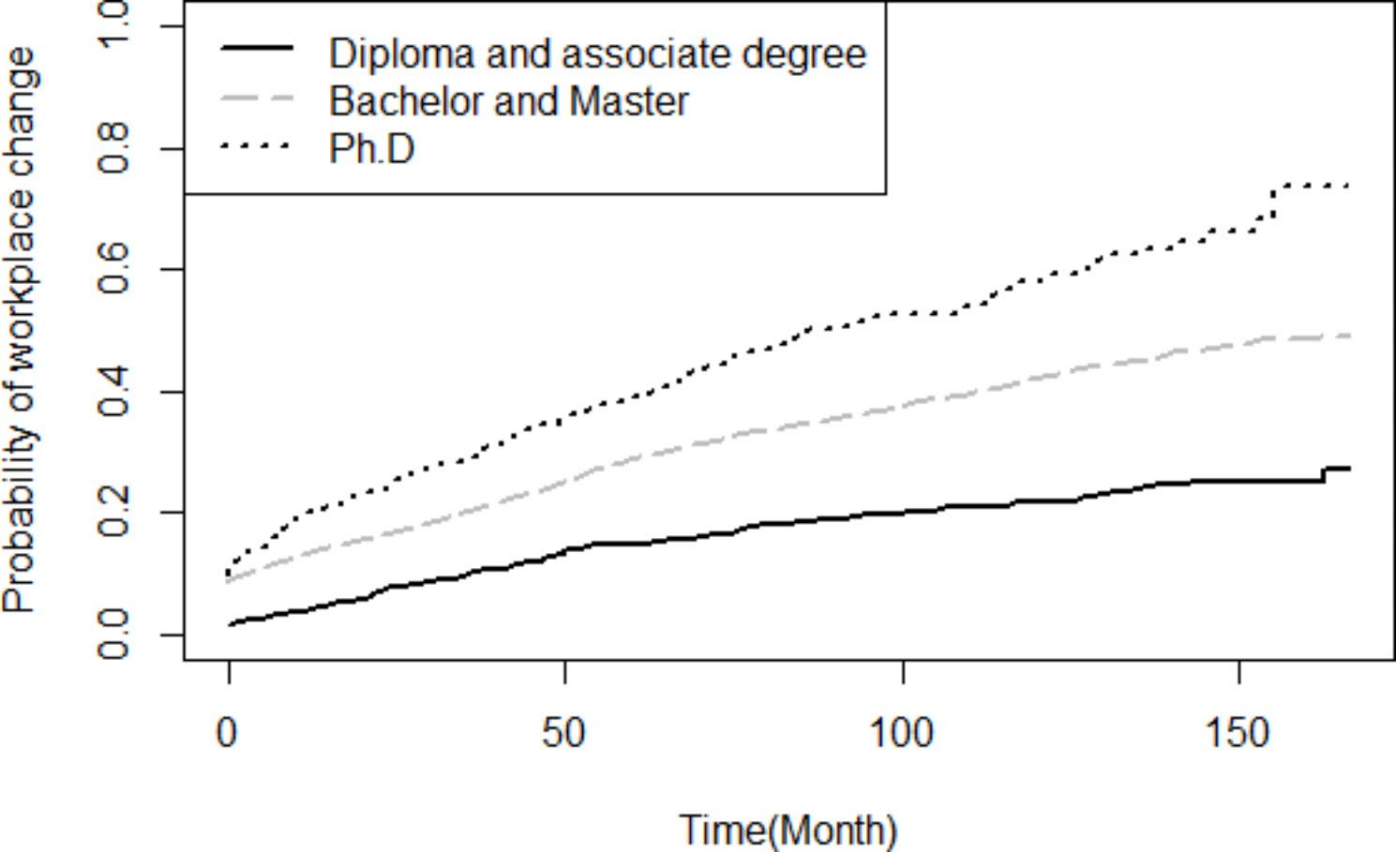
Comparison of the probability of turnover throughout the study period based on education

Based on the findings of the Log-rank test, the probability of turnover throughout the study period was not statistically different between male and female (p = 0.573), and likewise between employees with healthcare occupations and non-healthcare occupations (p = 0.351). Nevertheless, the probability of turnover was significantly higher for singles compared to married employees (p < 0.001) (Fig. 1). Employees whose current workplace and birthplace were not similar had a significantly higher probability of turnover (p < 0.001) (Fig. 2). Higher educated had more probability of turnover compared to lower educated (p < 0.001) (Fig. 3).

To simultaneously investigate the association between marital status and education variables and workplace retention (p < 0.2, Log-rank test), the Cox regression model was used. Based on the forward analysis method, marital status, education level, workplace and birthplace correspondence were selected in the final model.

Cox regression result showed, the risk of workplace change for the singles was 1.22 times higher than the married (p < 0.001), for the Ph.D degree was 3.23 times higher compared to those with a diploma or an associate degree (p < 0.001), and for a bachelor’s or master’s degree was 2.06 times more likely to change their workplace compared to diploma or an associate level of degree (p < 0.001) (Table 4).

**Table 4:** Analysis of the factors associated with probability of turnover using the Cox model

Variables		HR^*^	The 95% confidence interval for HR	p
Marital status	single	1.22	1.09–1.37	< 0.001
	Married (Ref)	1	-	-
Education level	PhD	3.23	2.63–3.97	< 0.001
	Bachelors & Masters	2.06	1.77–2.41	< 0.001
	Diploma & Associate degree (Ref)	1	-	-
workplace and birthplace correspondence	Yes	0.67	0.61–0.73	< 0.001
	No (Ref)	1	-	-

* Hazard Ratio

## Discussion

The purpose of our study was to examine the factors related to the non-retention and turnover of newly hired employees in the healthcare organizations under the jurisdiction of Mashhad University of Medical Sciences. Over the research period, Mashhad University of Medical Sciences experienced an attrition (turnover) rate of 31% for newly hired employees.

Survival analysis showed the probability of turnover for a newly recruited individual within one year, two years, and five years of employment was 0.12, 0.16, and 0.27 respectively and 69% of newly hired employees had never changed their workplace. The one year turnover rate of 12% for newly hired employees in this study was lower than the figure reported by Kim & Kim [29] for newly hired nurses at South Korean Hospitals (turnover rate: 26.4%) and Park and ko [30] (turnover rate:22%). The two year turnover rate observed in this study was similar to the findings of other research with a turnover rate of 17.7% that examined factors related to turnover of new graduate nurses between the years 2006–2008 [31]. In a longitudinal study by Hyoung Eun Chang and Sung-Hyun Cho, newly licensed nurse retention at the first job at one, two, and three years were 0.927, 0.778, and 0.686 respectively [32]. Consistent with prior studies, the results of our study indicate that the turnover rate of newly hired employees has increased over the studied period.

The disparities in turnover rates among studies may be linked to the labor market conditions in the regions in which the studies are conducted at the time of the research, as well as the occupational categories studied. This study shows that the turnover rate of newly hired employees was greater within the first year of employment rather than in subsequent years. Other studies which examined the tendency of turnover in newly hired nurses indicated that turnover intentions grows dramatically at 5 months of employment [33, 34]. It can be inferred that employees participate in employment tests, regardless of the prevailing conditions of the workplace, and may leave the organization shortly after joining it. Therefore, identifying the real reasons for leaving the organization of newly employed staff is necessary to design sustainability strategies.

This study found a no significant disparity between the probability of remaining at work and gender groups. This was in line with the findings of Al-Muallem and Al-Surimi [3], Blegen et al. [35] And Milles et al. [36], who found no significant association between gender and desire to leave their workplace in their investigations. However, according to Kim and Lee (2017), male nurses were more likely than female nurses to abandon their jobs [37]. Three likely reasons can be considered for discrepancy in findings between studies. First, the findings may be reflecting a gender imbalance in the research population. Second, in this study, all newly hired employees were evaluated, whereas, in previous studies Kim and Lee (2017) study was restricted to nurses. Third, this study was for 50 months duration, but the previous studies were typically shorter. The marital status of individuals and the workplace retention was observed to have a significant association in this study, as the single individuals were most likely to change their workplace than the married, consistent with the figures reported by Worku et al.(2019) and de Oliveira et al. (2017), all reported similar results [38, 39]. This might be because the single employees, in contrast of married are freer to move anywhere without worry, get better job opportunities and favorable labor market. Furthermore, marriage might enhance individual’s family and social responsibilities, which leads to a reduction in the inclination of married to leave the existing workplace.

The educational level was a factor significantly associated with the probability of turnover among new employees. Individuals with a higher level of education were more likely to quit their jobs. This is congruent with the findings of Lee’s (2019) and other research [40]. Individuals with higher education may have higher expectations and aspirations. Failing to meet their expectations and lacking an appropriate incentive system for higher education may be both discouraging and enhancing their one’s willingness to leave their existing workplace for better opportunities.

This study concluded that employees whose current workplace and their birthplace was not similar had a strikingly higher risk of workplace change which was consistent with the results of the study by Mohammadiaghdam et al. (2020) and Mollaei et al. (2021) [41, 42]. Those who live with their families and relatives benefit from family support, urban lifestyle and more facilities in workplace than those who have commuting issues, isolation, loneliness, and homesickness. It leads that native employees are less likely to leave existing workplace.

## Study limitations

Despite the notable findings, this study, like most others, had limitations. Collecting required accurate information was a time-consuming process which was resolved by support of human resource management of the MUMS. Due to incomplete records and information on date of employee resignation request were not accessible to this study and hence, we used the available information on the employees’ leaving dates. This constraint can be overlooked since the average time between requesting a resignation and leaving the workplace is two months in Iran. Another limitation of this study was the wide variety of healthcare professions and titles, which could make the statistical analysis more challenging. This was solved by putting all healthcare occupations into a “health field” category. Another limitation of this study was the lack of information about an employee`s occupational disease.

## Conclusion

The high turnover of professional staff leads to a decrease in the budget required to provide health care services delivery due to additional recruitment costs, and job vacancies caused by the turnover can pose occupational safety risks for the other employees due to increased workload. High level of employee turnover limits the ability of healthcare organizations to provide quality services. It is therefore imperative to consider the factors affecting the increased turnover of health workers. Policies promoting the recruitment of native-born professionals, the priority to married candidates than singles, and/or requiring single candidates to pledge to stay in the locality recruitment site can lead to increase the staff retention and reduce the costs of re-hiring including initial and on-the-job training, accommodation, other extra living consumptions and concern being away from home and family.

In light of this study findings, we suggest that employment policies should be revised to reduce employee turnover. A comprehensive analysis of the factors associated with the turnover of newly hired employees in different job positions may be valuable in future studies to obtain a purposeful understanding of the issue.

## Electronic supplementary material

Below is the link to the electronic supplementary material.

Supplementary Material 1

## Data Availability

The datasets generated and/or analyzed during this study are not publicly available confidentiality of information but are available from the corresponding author on reasonable request basis.

## Abbreviations

MUMS: Mashhad University of Medical Sciences
SST: Seyed Saedd Tabatabaee
VGH: Vahid GHavami
