# Supplementary material for: A survival analysis approach to determine factors associated with non-retention of newly hired health workers in Iran
Source: BMC Health Serv Res. 2023 Mar 16;23:265. doi: 10.1186/s12913-023-09262-5 (PMC10022210; doi:10.1186/s12913-023-09262-5)
Supplement: Supplementary file 1 — Supplementary Material 1 [file 12913_2023_9262_MOESM1_ESM.docx]

Additional file 1: The frequency of the job position

| Job position | Frequency |
| --- | --- |
| Operator | 1 |
| Stock clerk | 1 |
| librarian | 1 |
| Behvarz | 356 |
| Nurse assistant | 9 |
| Nurse | 3064 |
| General practitioner | 19 |
| General practitioner | 132 |
| Specialist practitioner | 135 |
| cleaner | 1 |
| System analyzer | 20 |
| Accountant | 159 |
| Pharmacist | 19 |
| Dentist | 11 |
| Psychologist | 6 |
| Physiotherapist | 4 |
| Lab technician | 19 |
| Diagnosis Lab technician | 45 |
| Operating room technician | 86 |
| Emergency medical technician | 209 |
| Professional health technician | 3 |
| Family health technician | 86 |
| Dental hygienist | 1 |
| Environmental health technician | 23 |
| Health technician | 4 |
| Medical records technician | 17 |
| Radiotherapy technician | 1 |
| Radiology technician | 22 |
| Disease prevention technician | 40 |
| Radiology technician | 13 |
| Health information technology technician | 15 |
| Medical records technician | 2 |
| Anesthesia technician | 33 |
| Lab expert | 287 |
| Statistics expert | 11 |
| Operating room expert | 123 |
| Health economic expert | 1 |
| Educational expert | 7 |
| Organizational health expert | 18 |
| Research expert | 1 |
| Students expert | 4 |
| Speech Therapy | 5 |
| Official expert | 41 |
| Hospital affair expert | 1 |
| Contracts expert | 2 |
| pharmacist | 14 |
| Psychologist | 8 |
| Information technology expert | 3 |
| Planning expert | 5 |
| Sports expert | 2 |
| Emergency medical expert | 190 |
| electrician | 2 |
| Budget expert | 7 |
| Professional health expert | 18 |
| Family health expert | 203 |
| Mental addiction health expert | 4 |
| Environmental health expert | 103 |
| Optometry expert | 5 |
| medical records expert | 52 |
| Radiotherapy expert | 8 |
| Radiology expert | 61 |
| Biomedical engineering | 17 |
| System analyzer expert | 12 |
| Nutrition expert | 28 |
| Rehabilitation expert | 20 |
| Data collection expert | 2 |
| Educational expert | 5 |
| Radiology expert | 38 |
| Radiology expert | 4 |
| Public relations expert | 3 |
| Mental health expert | 11 |
| Audiologists | 2 |
| Health information technology expert | 158 |
| Disease prevention expert | 98 |
| Medical records expert | 8 |
| Food and Health expert | 3 |
| Food and Health expert | 3 |
| Food and Health expert | 3 |
| Anesthesia expert | 113 |
| Staff Expert | 77 |
| librarian | 20 |
| Nurse aid | 1 |
| Mental Health expert | 1 |
| Midwife | 269 |
| security guard | 1 |
| Gynecologist | 1 |
| Clerk | 10 |
| Social worker | 22 |
| Financial administrative manager | 1 |
| Health mentor | 8 |
| Emergency Medical Services manager | 17 |
| Receptionist | 17 |
| Official services manager | 13 |
| Financial services manager | 53 |
| secretary | 12 |
| Selection manager | 3 |
| secretary | 1 |
| HVAC* | 4 |
| Civil Engineer | 7 |
| Guard | 2 |
| Total | 6811 |

* Heating, Ventilation, Air-Conditioning,
